# Supplementary material for: Mediating Effects of the COVID-19 Pandemic on the Associations between Physical Activity and Physical Fitness; Cross-Sectional Study among High School Adolescents
Source: J Funct Morphol Kinesiol. 2023 Sep 6;8(3):131. doi: 10.3390/jfmk8030131 (PMC10531934; doi:10.3390/jfmk8030131)
Supplement: Supplementary file 1 [file jfmk-08-00131-s001.zip › jfmk-2554180-supplementary.pdf]

Supplementary Table S1. Results of Kolmogorov Smirnov test (KS) for the total sample of participants

	Kolmogorov Smirnov test	
	Max D	*p*-value
Body height (cm)	0.12	$p < 0.10$
Body mass (kg)	0.09	$p < 0.15$
Body mass index	0.12	$p < 0.10$
Broad jump (cm)	0.08	$p > 0.20$
Sit-and-reach (cm)	0.08	$p > 0.20$
Sit-ups (number)	0.06	$p > 0.20$
Beep test (level)	0.07	$p > 0.20$
Step count	0.04	$p > 0.20$
